# Malarial Infection of Female BWF1 Lupus Mice Alters the Redox State in Kidney and Liver Tissues and Confers Protection against Lupus Nephritis

**DOI:** 10.1155/2013/156562

**Published:** 2013-11-10

**Authors:** Saleh Al-Quraishy, Mostafa A. Abdel-Maksoud, Azza El-Amir, Fathy A. Abdel-Ghaffar, Gamal Badr

**Affiliations:** ^1^Zoology Department, College of Science, King Saud University, Riyadh 11451, Saudi Arabia; ^2^Zoology Department, Faculty of Science, Cairo University, Cairo 61616, Egypt; ^3^Laboratory of Immunology & Molecular Biology, Zoology Department, Faculty of Science, Assiut University, Assiut 71516, Egypt

## Abstract

Systemic lupus erythematosus (SLE) is a prototypic autoimmune disease characterized by an imbalanced redox state and increased apoptosis. Tropical infections, particularly malaria, may confer protection against SLE. Oxidative stress is a hallmark of SLE. We have measured changes in the levels of nitric oxide (NO), hydrogen peroxide (H_2_O_2_), malondialdehyde (MDA), and reduced glutathione (GSH) in both kidney and liver tissues of female BWF1 lupus mice, an experimental model of SLE, after infection with either live or gamma-irradiated malaria. We observed a decrease in NO, H_2_O_2_, and MDA levels in kidney tissues after infection of lupus mice with live malaria. Similarly, the levels of NO and H_2_O_2_ were significantly decreased in the liver tissues of lupus mice after infection with live malaria. Conversely, GSH levels were obviously increased in both kidney and liver tissues after infection of lupus mice with either live or gamma-irradiated malaria. Liver and kidney functions were significantly altered after infection of lupus mice with live malaria. We further investigated the ultrastructural changes and detected the number of apoptotic cells in kidney and liver tissues in situ by electron microscopy and TUNEL assays. Our data reveal that infection of lupus mice with malaria confers protection against lupus nephritis.

## 1. Introduction

Systemic lupus erythematosus (SLE) is a multifactorial autoimmune disease that is characterized by the appearance of autoantibodies, particularly against nuclear components [1]. Because of its multifactorial etiology, which includes genetic, hormonal, and environmental triggers, the molecular mechanisms underlying this disease remain largely unknown. Free-radical-mediated reactions have recently drawn considerable attention as a potential mechanism of the pathogenesis of SLE [2]. Excessive generation of reactive oxygen species (ROS) has the potential to initiate damage to lipids, proteins, and DNA [3]. ROS represent a part of the defense mechanisms against microbes in the body. In addition, increased ROS generation has been reported to promote inflammation, necrosis, and apoptosis in chronic kidney disease [4]. Furthermore, previous studies have reported an association between oxidative stress and autoimmunity in an autoimmune-prone MRL+/+ mouse model [5]. Indeed, apoptosis plays an important role in SLE, and higher apoptosis rates lead to the production of autoantibodies, subsequently triggering disease activity [6]. The percentage of apoptotic cells in peripheral blood is significantly higher in SLE patients than in normal healthy patients, and the progression of human lupus nephritis is directly proportional to the increase in the rate of apoptosis [7]. Dysregulated apoptosis is believed to contribute to the development of SLE [8]. The delayed clearance of apoptotic cells induced by ROS production may prolong the interaction between cells, generating neoepitopes that subsequently cause broad-spectrum autoantibody formation, leading to tissue damage in SLE [9].

The levels of various cytokines are elevated in SLE patients and have therefore been considered essential elements in the etiopathology of the disease. IL-10 is an important immunoregulatory cytokine that inhibits T-cell function by suppressing the expression of proinflammatory cytokines [10]. Moreover, continuous administration of anti-IL-10 antibodies in a murine lupus model delayed the onset of autoimmunity and improved the survival rate from 10 to 80% [11]. TNF-*α* is a cytokine that is well known for its role in the regulation of inflammation and apoptosis, two processes involved in the pathogenesis of SLE. TNF-*α* is increased in SLE and is correlated with disease activity [12], and it has been proposed to contribute to the immunopathogenesis of SLE [13]. Conversely, previous findings showed diminished production of TNF-*α* in BWF1 mice associated with the development of severe disease manifestations, such as nephritis [14].

Tropical infections, particularly malaria, may confer protection against SLE [15]. Furthermore, epidemiological studies have revealed that SLE is rarely observed in rural tropical areas of Africa and Asia, where malarial infection is prevalent [16, 17]. Therefore, it has been hypothesized that SLE-susceptibility genes are beneficial in controlling severe malaria but promote inflammation in the absence of malaria [18, 19]. In this regard, Greenwood et al. described a higher survival rate in young lupus-prone mice infected with *Plasmodium berghei yoelii* [20]. Another study revealed that old BWF1 mice, when infected with *P. chabaudi* at the onset of clinical signs of lupus and subsequently treated with chloroquine, developed temporary remission of the symptoms. Moreover, the injection of immunoglobulins isolated from *P. chabaudi*-infected BALB/c mice produced similar protective effects as the infection itself in BWF1 mice [21]. Few studies have investigated the effect of malarial infection on SLE. Hence, this study aimed to investigate the possible role of malarial infection on oxidative stress, apoptosis, and cytokine levels induced by SLE in a murine lupus model. 

## 2. Materials and Methods

### 2.1. Animals

A total of 30 female BWF1 29-week-old mice were purchased from Jackson Laboratory (Bar Harbor, USA) and maintained, monitored, in a specific pathogen-free environment. All animal procedures were performed in accordance with the standards set forth in the Guidelines for the Care and Use of Experimental Animals by the Committee for the Purpose of Control and Supervision of Experiments on Animals (CPCSEA). The study protocol was approved by the Animal Ethics Committee at King Saud University. All animals were allowed to acclimatize in plastic cages (5 animals per cage) inside a well-ventilated room for 1 week prior to the experiment. The animals were maintained under standard laboratory conditions (temperature of 23°C, relative humidity of 60–70%, and a 12-hour light/dark cycle), fed a diet of standard commercial pellets, and given water *ad libitum*. 

### 2.2. Malarial Infection

The blood stage forms of *Plasmodium chabaudi* parasites were stored in liquid nitrogen after in vivo passage in 3-month-old BALB/c mice according to a previously described protocol [22]. Female BWF1 mice (30 weeks old) were infected by i.p. injection of 10^6^ parasitized erythrocytes obtained from an infected mouse of the same strain as previously described [21]. Parasitemia was monitored by Giemsa-stained thin blood smears. Experimental animals were assigned to 3 groups (10 mice/group) as follows: group (I) lupus group (lupus noninfected); group (II) live malaria-infected group (lupus + live malaria infection); and group (III) irradiated malaria-infected group (lupus + irradiated malaria-infection). Group III was infected i.p. with 10^6^ gamma irradiated red blood cells (iRBCs) infected with *P. chabaudi. *Prior to injection, the blood cells were exposed to a dose of 200 Gy gamma-radiation from a Gamma Cell 200 Irradiator (Atomic Energy of Canada, Ltd., Ottawa, Canada) utilizing a ^60^Co source located at the Research Center of College of Science, King Saud University, Saudi Arabia. This radiation dose was applied based on experiments conducted by Ferreira-da-Cruz et al. [23] that provided evidence that a 200-Gy gamma-irradiation dose is able to abolish the original replication of erythrocytic forms of the Palo Alto *P. falciparum *strain, most likely by inactivating their infectivity. According to their data, 100 or 150 Gy irradiation doses were unable to inactivate the parasite, despite the reduction of parasitemia. All animals were sacrificed at day 14 after infection.

### 2.3. Sample Collection

Blood was collected from the heart in heparinized tubes for the determination of hematological parameters and to obtain plasma for biochemical studies. Plasma was stored at −80°C until use. The liver and kidneys were removed and cut into small pieces in sterile saline. The pieces were fixed in glutaraldehyde for transmission electron microscopy or 4% paraformaldehyde for TUNEL assays or suspended in Tris buffer for biochemical studies.

### 2.4. Electron Microscopy

Small pieces of the liver and kidney tissues were fixed in 2.5% glutaraldehyde for 24 h. The small pieces were washed with phosphate buffer (0.1 M, pH 7.4). Postfixation was performed in 1% osmium tetroxide buffered to pH 7.4 with 0.1 M phosphate buffer at 4°C for 1-2 h and then washed again in phosphate buffer to remove the excess fixative. The samples were dehydrated in ascending grades of ethanol followed by clearing in propylene oxide. The specimens were embedded in araldite. Polymerization was achieved by incubating the capsules at 60°C. Ultrathin sections (100 nm) were prepared with an ultramicrotome and picked up on uncoated copper grids. Following double staining with uranyl acetate and lead citrate, the sections were examined and photographed using a JEOL 100 Cx transmission electron microscope (Japan).

### 2.5. TUNEL Assay

Liver and kidney tissues were fixed in 4% paraformaldehyde for 4 h at 4°C, washed in phosphate-buffered saline (PBS), and embedded in paraffin blocks. Sections of 7 *μ*m were cut and air-dried for 20 minutes before staining. Terminal dUTP nick-end labeling (TUNEL) using a commercial TUNEL Apoptosis Detection Kit FITC-labeled POD (Gen Script, USA) was performed according to the manufacturer's instructions. Apoptotic cells were identified by morphological criteria (cell shrinkage and chromatin condensation and margination). The apoptotic nuclei were stained dark brown, examined, and counted under a light microscope.

### 2.6. Cell Blood Count (CBC)

Whole blood samples were analyzed with an automatic Vet abc Animal Blood Counter (Horiba ABX, Montpellier, France) using the hematology kits specified for that instrument (Horiba ABX, France) according to the manufacturer's instructions.

### 2.7. Kidney and Liver Function Testing

Plasma samples were analyzed using commercial kits (bioMerieux, Marcy I'Etoile, France) for alanine aminotransferase (ALT), aspartate aminotransferase (AST), and creatinine (Creat.) according to the instructions of the manufacturer. Absorbance was measured with an Ultrospec 2000 U/V spectrophotometer (Amersham Pharmacia Biotech, Cambridge, England).

### 2.8. Oxidative Stress Assessment in Liver and Kidney Tissues

Oxidative stress markers were measured in liver and kidney homogenates whereas parts of liver and kidney were weighed and homogenized immediately to give 50% (w/v) homogenate in ice-cold medium containing 50 mM Tris-HCl and 300 mM sucrose [24]. The homogenate was centrifuged at 500 g for 10 min at 4°C. The supernatant (10%) was used for determination of NO, H_2_O_2_, MDA, and GSH using commercial kits (Biodiagnostic, Dokki, Giza, Egypt).

Nitrite/nitrate is assayed according to the technique of Berkels et al. [25]. In brief, nitrous acid is formed in acid medium, and in the presence of nitrite, the formed acid diazotizes sulphanilamide, which is coupled with N-(1-naphthyl)ethylenediamine, and the resulting azo dye is measured at 540 nm.

Lipid peroxidation is determined by the method of Ohkawa et al. [26]. Homogenate is suspended in 1 mL of 10% trichloroacetic acid and 1 mL of 0.67% thiobarbituric acid boiled in a water bath for 30 min. Thiobarbituric acid reactive substances are measured at 535 nm and expressed as malondialdehyde (MDA) equivalents formed.

Hydrogen peroxide (H_2_O_2_) is assayed according to Aebi [27]. In the presence of horse radish peroxidase (HRP), H_2_O_2_ in tissue homogenate reacts with 3,5-dichloro-2-hydroxybenzenesulfonic (DHBS) acid and 4-aminophenazone (AAP) to form a chromophore that can be quantified at 240 nm.

Glutathione (GSH) is measured with Ellman's reagent [28]. This reagent is reduced to produce a yellow chromogen, which is directly proportional to the GSH concentration which is measured at 405 nm.

### 2.9. Measurement of TNF-*α* and IL-10

Plasma levels of TNF-*α* and IL-10 were measured in duplicate using purified biotinylated antibodies in ELISA sets according to the protocol provided by the supplier (Abcam, Cambridge, UK). The ELISA plates were read with a microplate reader (Multiskan ASCENT ThermoH).

### 2.10. Statistical Analysis

The data were tested for normality using the Anderson-Darling test and for homogeneity variances prior to further statistical analysis. The data were normally distributed and are expressed as the mean ± standard error of the mean (SEM). Significant differences among the groups were analyzed by one- or two-way ANOVA followed by Bonferroni's test for multiple comparisons using PRISM statistical software (GraphPad Software). The data were also reanalyzed by one- or two-way ANOVA followed by Tukey's posttest using SPSS software, version 17. Differences were considered statistically significant at *P* < 0.05.

## 3. Results

### 3.1. Live Malarial Infection Decreases Oxidative Stress Markers Induced by SLE in Renal and Hepatic Tissues

**Figure 1:**
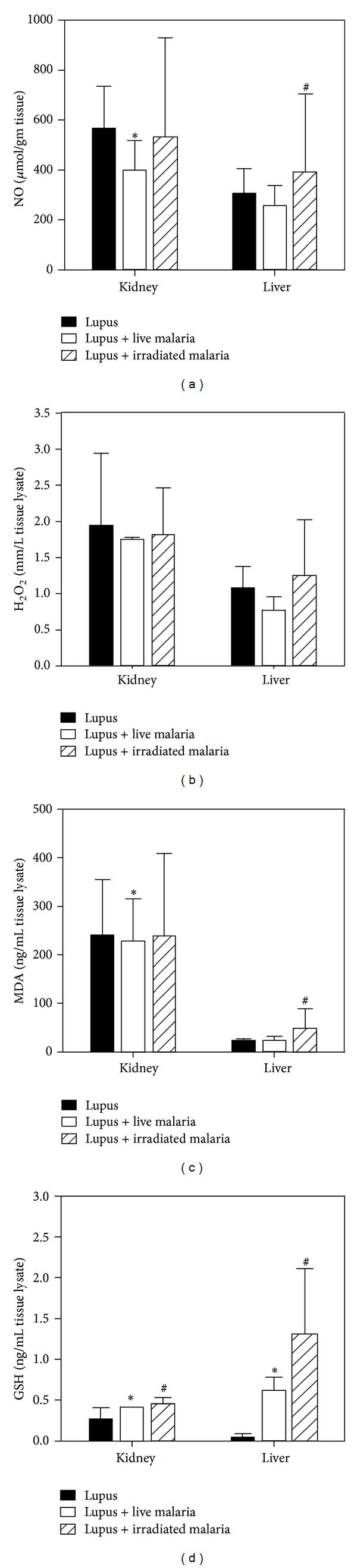
Effect of live or gamma-irradiated malarial infection on renal and hepatic NO, H_2_O_2_, MDA, and GSH levels. The levels of NO (a), H_2_O_2_ (b), MDA (c), and GSH (d) in kidney and liver tissue of BWF1 lupus mice after experimental infection with either live or gamma-irradiated malariain comparison with the lupus noninfected group. The data are the mean ± SEM for 6 mice per group for the live malaria-infected group (open white bars), gamma-irradiated malaria-infected group (hatched bars), and lupus noninfected group (closed black bars). *P < 0.05 for live malaria-infected BWF1 mice versus lupus; ^#^ *P* < 0.05 for gamma-irradiated malaria-infected BWF1 mice versus lupus mice; ^+^ *P* < 0.05 for live malaria-infected BWF1 mice versus gamma-irradiated malaria-infected BWF1 mice.

The effect of malarial infection on oxidative stress markers in the SLE experimental model was measured by determining the levels of NO, H_2_O_2_, and MDA in kidney and liver samples of the three experimental groups of female BWF1 mice, whereas GSH was the only antioxidant parameter measured in these tissues. We observed a significant decrease in the levels of NO, H_2_O_2_, and MDA (Figures 1(a), 1(b), and 1(c)) in renal and hepatic tissues after live malarial infection compared with lupus mice (*P < 0.05), whereas the GSH concentration was increased after live or gamma-irradiated malaria infection (Figure 1(d)). However, gamma-irradiated malarial infection resulted in diminished levels of NO, H_2_O_2_, and MDA in renal tissue compared with lupus mice (^#^ *P* < 0.05). By contrast, in hepatic tissue, gamma-irradiated malarial infection had a different outcome, with higher levels of NO, H_2_O_2_, MDA, and GSH than those observed in lupus mice.

### 3.2. Altered Blood Cell Numbers after Malarial Infection in BWF1 Mice

Infection of BWF1 mice with malaria (either live or gamma-irradiated) had an obvious effect on blood cell numbers. The erythrocyte count decreased significantly after infection with live malaria (*P < 0.05) compared with lupus mice (Table 1). However, the total leukocyte counts were highly increased in both the live and gamma-irradiated malarial infection groups, with a larger increase in the live infection group (9.98 ± 0.89) than in the gamma-irradiated group (7.10 ± 0.34). There was an obvious infection-related effect on liver weights. We observed a significant increase in liver weights in the live malaria-infected group (*P < 0.05), whereas the liver weights of the gamma-irradiated malaria-infected group did not clearly change. Kidney weights were slightly reduced in the live malaria-infected group. Spleen weights were less clearly affected by infection with either live or gamma-irradiated malaria than liver weights (data not shown).

### 3.3. Malarial Infection Has No Ameliorating Effect on Either Kidney Or Liver Functions in BWF1 Mice

**Figure 2:**
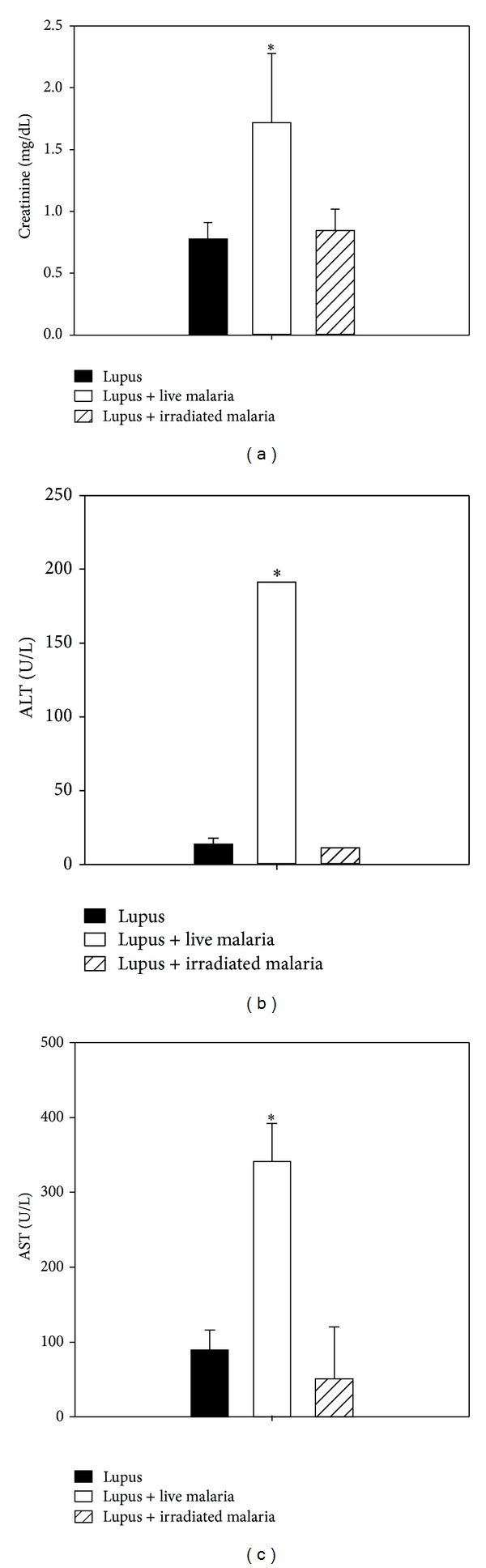
The effect of malarial infection on kidney and liver function. The effect of malaria on kidney and liver function in BWF1 mice. (a) Creatinine concentration (mg/dL). (b) ALT concentration (U/L). (c) AST concentration (U/L) in plasma of all groups. The data are the mean ± SEM for 6 mice per group for the live malaria-infected group (open white bars), gamma-irradiated malaria-infected group (hatched bars), and lupus noninfected group (closed black bars). *P < 0.05 for live malaria-infected BWF1 mice versus lupus mice; ^#^ *P* < 0.05 for gamma-irradiated malaria-infected BWF1 mice versus lupus mice; ^+^ *P* < 0.05 for live malaria-infected BWF1 mice versus gamma-irradiated malaria-infected BWF1 mice.

Kidney function in BWF1 mice, as evaluated by estimating creatinine levels in plasma samples from lupus mice, was impaired; however, live malaria infection had no effect on this impairment (Figure 2(a)). By contrast, liver function, as measured by ALT (Figure 2(b)) and AST (Figure 2(c)) concentrations in plasma samples of the three groups, was severely impaired in the live malaria-infected group compared with either the lupus or gamma-irradiated malaria-infected groups.

### 3.4. Live Malaria Infection Alleviates Renal Cytopathological Signs of Lupus

**Figure 3:**
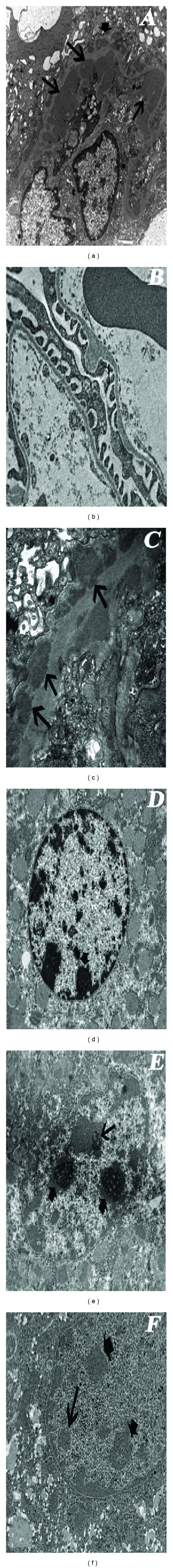
Kidney and liver cytopathological features after malarial infection. Effect of malaria on renal and hepatic cytopathology. Kidney and liver sections were taken from BWF1 mice at week 32. TEM photomicrographs of the kidneys showed electron-dense deposits (arrows) in both the lupus and gamma-irradiated malaria-infected groups ((a), (c)) with marked effacement of foot processes (arrow head). The live malaria-infected group showed normal foot processes with an absence of deposits (b). The liver of a lupus mouse showing a normal structure of a hepatocyte with its nucleus, mitochondrion, and rough endoplasmic reticulum (d). Liver section from the live malaria-infected group showing swollen heterochromatic nuclei and disrupted chromatin (arrow) (e). Liver section from the gamma-irradiated malaria-infected group showing vacuolated and hypertrophic nucleoli (arrow head) (f). Original magnification 10000x.

In the electron microscopic observations, renal tissue sections of lupus mice displayed massive mesangial, subendothelial, and subepithelial deposits; diffused effacement of foot processes; mesangial and endocapillary proliferation; and occlusion of several capillary lumens (Figure 3(a)). The kidney sections of the live malaria-infected group displayed normal foot process width and nearly complete absence of dense deposits (Figure 3(b)). In the gamma-irradiated malaria-infected group, there were subendothelial and subepithelial deposits with diffused effacement of foot processes (Figure 3(c)).

In hepatic tissue,the characteristic apoptotic features of hepatocytes, such as chromatin condensation, vacuolation and hypertrophy of nucleoli, and membrane blebbing, were observed in the hepatocytes of malaria-infected groups (either live or gamma irradiated) (Figures 3(e) and 3(f)) but were not present in lupus noninfected livers (Figure 3(d)).

### 3.5. Apoptotic Changes in the Renal and Hepatic Tissues of Lupus Mice before and after Malarial Infection

**Figure 4:**
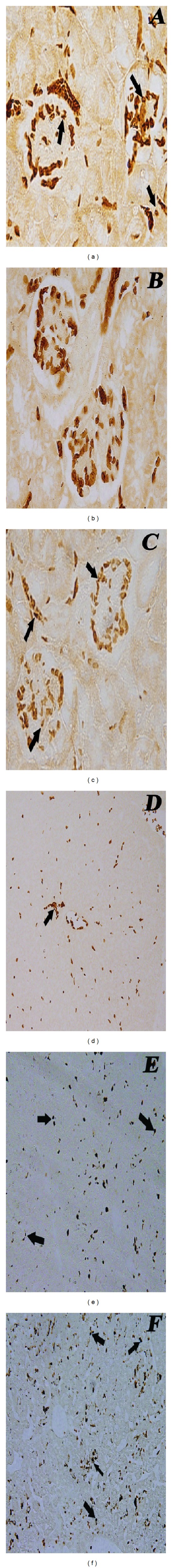
Apoptotic changes in renal and hepatic tissues after malaria infection. The effect of malarial infection on apoptotic DNA fragmentation in renal and hepatic tissues of female BWF1 mice. Kidney tissues of the lupus (a), live malaria (b), and gamma-irradiated (c) infected group and liver tissues of the lupus (d), live malaria (e), and gamma-irradiated (f) infected group of BWF1 mice at week 32. Paraffin-embedded tissue sections were prepared and investigated using TUNEL apoptosis detection kits. The TUNEL-positive nuclei (arrows) are markedly different from those observed in the lupus group. Original magnification, 1000x in the kidney tissue, and 400x in the live tissue.

Kidney and liver sections were stained using TUNEL apoptosis detection kits to confirm the apoptotic changes in both renal and hepatic tissues. We observed that kidney sections from the live malaria-infected group exhibited fewer apoptotic cells (Figure 4(b)) compared with the lupus group or the gamma-irradiated malaria-infected group (Figures 4(a) and 4(c)). By contrast, liver sections from lupus mice displayed the fewest apoptotic cells (Figure 4(d)), and there was a significant increase in apoptotic cells in the livers from the malaria groups (either live or gamma irradiated) (Figures 4(e) and 4(f)). The results of the TUNEL evaluation are presented in Table 2.

### 3.6. Effect of Live Malarial Infection on Plasma Levels of TNF-*α* and IL-10

**Figure 5:**
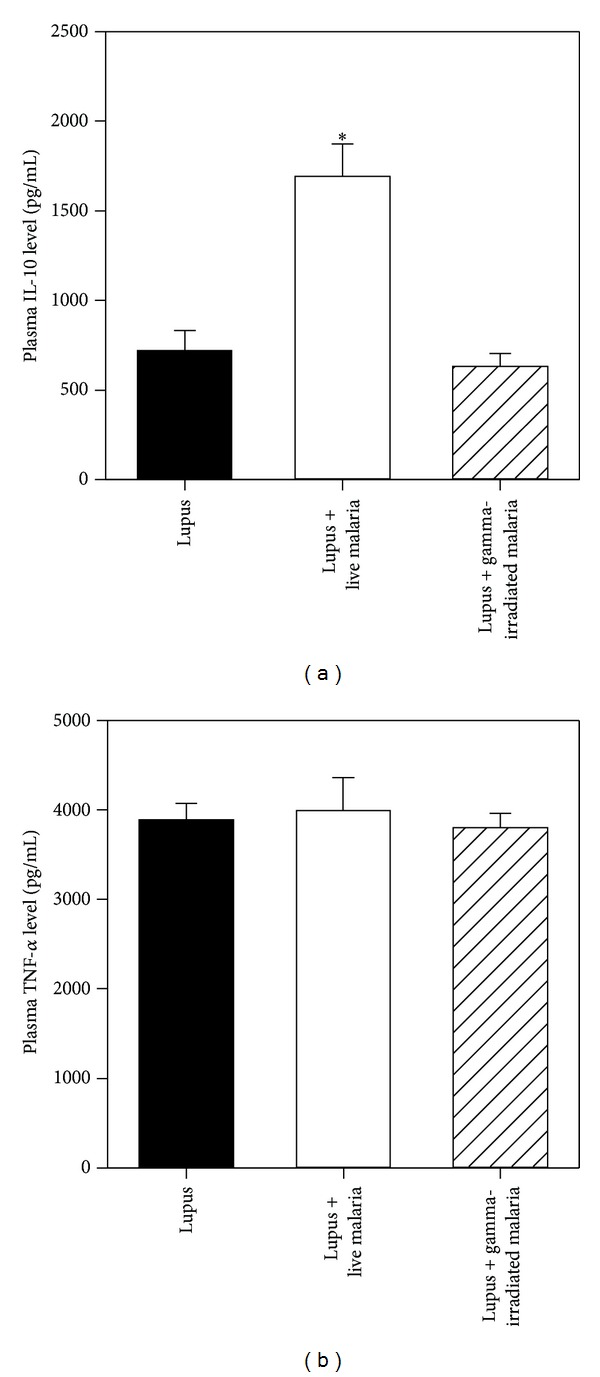
Alterations in the plasma levels of TNF-*α* and IL-10 cytokines after malarial infection. The levels of plasma pro-inflammatory (TNF-*α*) and anti-inflammatory (IL-10) cytokines were measured in the three groups of mice by ELISA. The results are presented as the cytokine levels (pg) per mL of plasma and are expressed as the mean ± SEM (*n* = 10). *P < 0.05 for live malaria-infected BWF1 mice versus lupus mice; ^#^ *P* < 0.05 for gamma-irradiated malaria-infected BWF1 mice versus lupus mice; ^+^ *P* < 0.05 for live malaria-infected BWF1 mice versus gamma-irradiated malaria-infected BWF1 mice.

We compared the plasma TNF-*α* and IL-10 concentrations in malaria-infected groups with those in the lupus group. The accumulated data from 10 individual mice from each group, which are shown in Figures 5(a) and 5(b), indicated a significant increase (*P < 0.05) in the plasma levels of TNF-*α* and IL-10 in the live malaria-infected group compared with the lupus noninfected group, whereas the levels of both cytokines in the gamma-irradiated malaria-infected group were similar to those of the lupus group, illustrating that a viable parasite is essential for the observed malaria-ameliorating effect. 

## 4. Discussion

SLE is a chronic inflammatory autoimmune disease of multiple origins. There is accumulating evidence that the increase in autoimmune diseases observed in Western countries is partly caused by a decline in infectious diseases [29]. For example, it has been observed that rotavirus infection in infant and young adult nonobese diabetic mice delays the onset of diabetes and reduces its incidence [30]. Here, we investigated the effect of the injection of female BWF1 mice with either live or gamma-irradiated malaria on the oxidative stress and apoptosis induced by SLE. Our results clearly indicate an ameliorating effect of live malaria on renal tissue through restoration of GSH levels and decreases in the levels of NO, H_2_O_2_, and MDA, but this effect was unclear in the gamma-irradiated malaria group. A recent study has shown that *Toxoplasma gondii* infection may prevent the development of a lupus-like syndrome in autoimmune BWF1 mice [31]. The severity of disease was positively associated with increased levels of intracellular ROS production and lipid peroxidation in SLE mice, in accordance with the reports of Tewthanom et al. [32] and Túri et al. [33], which demonstrated that increased oxidative stress was correlated with active glomerular disease in SLE patients. Adequate concentrations of glutathione are required for a variety of functions, including protection of the cell from oxidative damage by quenching of oxidant species, lymphocyte activation, natural killer cell activation, and lymphocyte-mediated cytotoxicity [34]. Decreased intracellular GSH levels may be due to ROS-induced GSH oxidation or GSH export from cells. The resultant GSH reduction would enhance further ROS production during oxidative challenge [35]. A compromised antioxidant defense system has been implicated in both the development and acceleration of lupus nephritis. In the present study, the decrease in the levels of intracellular GSH in lupus mice was negatively correlated with the severity of disease, as confirmed by TEM. However, malarial infection increased the levels of GSH in both renal and hepatic tissues. Kidney function was not significantly improved after malarial infection, in agreement with a study by Greenwood et al. [20], who reported a slight decrease in kidney function postmalaria infection. It appears that 14 days is not enough time to restore the impaired kidney function in lupus mice and, instead, chronic malarial infection is needed. The antiapoptotic effect of malaria was observed in TUNEL photomicrographs as a decrease in the number of apoptotic nuclei in the renal cells of the live malaria-infected group. Indeed, renal oxidative stress is a characteristic feature of both SLE and malaria infection. However, it seems that oxidative stress parameters that cause renal tissue damage in lupus are directed towards antiparasitic defense during malaria infection. In healthy (nonlupus) mice Elias et al. have reported that products of oxidative stress as well as the immune response against the parasite are crucial to changes in kidney architecture (malaria-associated-acute kidney injury) and microvascular endothelial permeability of BALB/c mice infected with *P. berghei* ANKA [36]. In hepatic tissue, however, malarial infection significantly induced liver apoptosis. The apoptosis of hepatocytes during malarial infection is well correlated with liver dysfunction, as indicated by liver function testing. Kochar et al. (2003) reported hepatic dysfunction in malarial infection and histopathological changes such as hepatocyte necrosis, cholestasis, granulomatous lesions, and malarial nodules [37]. The hepatomegaly observed in the live malaria-infected group is in concordance with other reports [38]. Our results agree with the results of Shah et al. [4], who reported that increased apoptosis of T lymphocyte subsets may be mediated by decreased intracellular glutathione concentrations and that the severity of disease might be enhanced by overproduction of ROS in SLE. The decrease in RBCs and the increase in WBCs is a well-known clinical manifestation of malarial infection. Our results regarding gamma-irradiated malaria are interesting because the high dose of gamma radiation used in this study was sufficient to kill the parasite, resulting in a group that resembled the lupus noninfected group in the kidneys whereas, in the liver, the gamma-irradiated malaria-infected group fared worse than either the lupus or live malaria-infected groups. The different outcomes in these two organs require further investigation. Cytokine imbalance plays a significant role in the acceleration of lupus-like autoimmune disease. In this study, we observed that live malarial infection increased plasma levels of TNF-*α* and IL-10 cytokines. The manipulation of these cytokines may represent a potential therapeutic strategy for the treatment of SLE [39]. Previous studies have shown that TNF-*α* deficiency is an important trigger and driver of lupus-like autoimmunity in NZB/W mice [40]. In addition, it has been reported that over-expression of IL-10 in lupus-prone NZM2410 mice can ameliorate lupus diseases [41].

Taken together, our data reveal an ameliorating effect in the renal tissue of female BWF1 mice at both the structural and functional levels after live malarial infection.

## 5. Conclusion

Our data demonstrate that malaria infection alleviates lupus nephritis by decreasing renal oxidative stress and increasing the antioxidant defense system.

## Figures and Tables

**Table 1:** Blood cell counts and relative organ weights in lupus, live, and gamma-irradiated malaria-infected BWF1 mice.

Experimental group	Blood cell counts		Relative organ weights (g/100 g body weight)	
	RBC (10^6^/mm^3^)	WBC (10^3^/mm^3^)	Kidney	Liver
Lupus	6.83 ± 0.33	5.91 ± 0.21	1.89 ± 0.03	4.44 ± 0.16
Lupus + live malaria	4.17 ± 0.27*	9.98 ± 0.89*	1.70 ± 0.03	4.95 ± 0.08*
Lupus + gamma-irradiated malaria	6.75 ± 0.32	7.10 ± 0.34^#^	1.69 ± 0.04	4.39 ± 0.13

Mean ± SEM, *n* = 6. *P < 0.05 for live malaria-infected BWF1 mice versus lupus mice; ^#^ *P* < 0.05 for gamma-irradiated malaria-infected BWF1 mice versus lupus mice; ^+^ *P* < 0.05 for live malaria-infected BWF1 mice versus gamma-irradiated malaria-infected BWF1 mice.

**Table 2:** Incidence and number of apoptotic cells in renal and hepatic tissues of lupus, live, and gamma-irradiated malaria-infected BWF1 mice.

Experimental group	Number of apoptotic cells (cm^2^ in kidney)	Number of apoptotic cells (cm^2^ in liver)
Lupus	20 ± 5	3 ± 1
Lupus + live malaria	10 ± 3*	30 ± 7^∗#^
Lupus + gamma-irradiated malaria	19 ± 2	37 ± 4^∗#^

Mean ± SEM, *n* = 6. *P < 0.05 for live malaria-infected BWF1 mice versus lupus mice; ^#^ *P* < 0.05 for gamma-irradiated malaria-infected BWF1 mice versus lupus mice; ^+^ *P* < 0.05 for live malaria-infected BWF1 mice versus gamma-irradiated malaria-infected BWF1 mice.
